# CONCEPTUALIZING THE HEALTH CARE WORKFORCE AS A SOCIAL DETERMINANT OF HEALTH IN LONG-TERM CARE SETTINGS

**DOI:** 10.1093/geroni/igae098.3209

**Published:** 2024-12-31

**Authors:** Roy Thompson, Sherif Olanrewaju, Allison Squires

**Affiliations:** Hunter College, City University of New York, Philadelphia, Pennsylvania, United States; The Pennsylvania State University, University Park, Pennsylvania, United States; New York University, New York, New York, United States

## Abstract

The social determinants of health include environmental conditions where: individuals reside and age that affect their health, functioning, quality of life, and risks. We propose a conceptual model of how the health workforce becomes a social determinant of health resulting from the sustained person-clinician relationship. To illustrate the application of this proposed model, we examine the case of long-stay residents in long-term care settings. These residents spend ≥101 days, oftentimes until death in these facilities and have prolonged healthcare staff interactions which impact their health outcomes. Evidence suggests that sustained contact between residents and the healthcare workforce does impact their quality of life, health outcomes, and risks. We posit that the health workforce can be a contributing factor in inequitable outcomes in long-term care settings, thus becomes a social determinant of health. Their interactions and practices with residents may explain disparities in Black and Hispanic residents who typically have significantly higher rates of pain, falls, antipsychotic use, and pressure injuries relative to their Non-Hispanic White counterparts. Additionally, Black and Hispanic residents receive care in geographically segregated nursing homes. Through multi-level exemplars, we will illustrate how as a social determinant the workforce characteristics like racial and ethnic concordance, skill mix, and hours per resident impact health outcomes. Therefore, studies that conceptualize and operationalize the healthcare workforce as a social determinant of health at the individual, interpersonal and organizational levels are needed to improve staffing interventions to mitigate health disparities across in long-term care settings.

